# The Prognostic Impact of Tumor Location in pT3N0M0 Upper Urinary Tract Urothelial Carcinoma: A Retrospective Cohort Study

**DOI:** 10.3389/fonc.2022.850874

**Published:** 2022-03-15

**Authors:** Tzu Shuang Chen, Yen Ta Chen, Hung Jen Wang, Po Hui Chiang, Wen Chou Yang, Wei Ching Lee, Yao Chi Chuang, Yuan Tso Cheng, Chih Hsiung Kang, Wei Chia Lee, Chien Hsu Chen, Yuan Chi Shen, Yi Yang Liu, Hui Ying Liu, Yin Lun Chang, Yu Li Su, Chun Chieh Huang, Hao Lun Luo

**Affiliations:** ^1^ Department of Urology, Kaohsiung Chang Gung Memorial Hospital and Chang Gung University College of Medicine, Kaohsiung, Taiwan; ^2^ Jhong Siao Urological Hospital, Kaohsiung, Taiwan; ^3^ Department of Hematology and Oncology, Kaohsiung Chang Gung Memorial Hospital and Chang Gung University College of Medicine, Kaohsiung, Taiwan; ^4^ Department of Radiation Oncology, Kaohsiung Chang Gung Memorial Hospital and Chang Gung University College of Medicine, Kaohsiung, Taiwan

**Keywords:** tumor location, upper urinary tract urothelial carcinoma, oncological outcomes, pT3N0M0, radical nephroureterectomy

## Abstract

**Background:**

We aimed to evaluate the impact of tumor location on cancer outcomes in patients with pT3N0M0 upper tract urothelial carcinoma (UTUC) treated with radical nephroureterectomy (RNU) with bladder cuff excision.

**Materials and Methods:**

We retrospectively reviewed 302 patients with pT3N0M0 UTUC who underwent RNU with bladder cuff excision at our institution between 2005 and 2019, including 191 renal pelvis tumors and 111 ureteral tumors. Clinicopathologic characteristics were compared between renal pelvis and ureter urothelial carcinomas. Multivariate Cox proportional hazard regression was used to assess the association between outcomes and clinical factors. Outcomes of interest included intravesical recurrence-free survival (IVRFS), local recurrence-free survival (LRFS), distant metastasis-free survival (DMFS), and cancer-specific survival (CSS), which were measured using the Kaplan–Meier curve with a log-rank test.

**Results:**

A total of 302 patients underwent RNU with bladder cuff excision. During the median follow-up of 42.7 months, 70 (23.2%), 95 (31.5%), and 99 (32.8%) patients experienced intravesical recurrence, local recurrence, and distant metastasis, respectively. Seventy (23.2%) patients died from UTUC. Multivariate Cox regression analysis showed that tumor location was an independent predictor of local recurrence (HR = 2.05, p = 0.001), with borderline independent significance in intravesical recurrence (HR = 1.54, p = 0.074) and distant metastasis (HR = 1.45, p = 0.08). Kaplan–Meier analysis showed that ureter tumors had a worse 5-year local recurrence (log-rank p < 0.001) and borderline worse 5-year intravesical recurrence (log-rank p = 0.055) and 5-year distant metastasis (log-rank p = 0.073).

**Conclusion:**

Ureter tumors seem to be associated with worse oncological outcomes, especially with local recurrence in UTUC. Further large and long-term studies are warranted for investigating biological differences based on tumor location.

## Introduction

Urothelial carcinoma (UC), the fourth most common malignancy worldwide, can be located in the upper (pyelocaliceal cavity and ureter) or lower (bladder and urethra) urinary tract. Upper urinary tract urothelial carcinoma (UTUC) is uncommon and accounts for only 5% of all urothelial tumors (1). The estimated annual incidence of UTUC is 2/100000 in Western countries. Most cases occur in the renal pelvis and are approximately twice as common as ureteral tumors. For decades, radical nephroureterectomy (RNU) with bladder cuff excision has been regarded as the standard treatment for patients with UTUC. Furthermore, segmental resection and endoscopic management can also be considered in selected patients based on tumor location, tumor size, and histological characteristics (2).

In Taiwan, UTUC has an unusually high incidence and female predominance. It also accounts for >30% of UC cases in the country according to the Taiwan Cancer Registry Annual Report in 2017. Previous reports have demonstrated that cigarette smoking, arsenic exposure, and occupational carcinogens lead to this phenomenon (3, 4). Due to scant symptoms and delayed diagnosis, UTUC presented at least a muscle-invasive appearance (56%), which results in worse outcomes than those of bladder cancer (5). Patients with advanced UTUC tend to have a poor prognosis and high recurrence rates after RNU (1, 6). Therefore, identifying potential risk factors for UTUC is an important public health issue.

Tumor stage, grade, and lymphovascular invasion are the most significant prognostic factors in patients with UTUC (2). However, whether tumor location is an independent predictor of oncologic outcomes remains controversial. Several studies have reported that ureteral lesions are significantly associated with worse recurrence-free survival (RFS). Moreover, ureteral tumor is an independent risk factor that may result in worse cancer-specific death, recurrence, and metastasis in patients with UTUC (7, 8). Tumors invading the peripelvic or periureteral fat are associated with an approximately 3.5 times higher risk of cancer-specific death than tumors invading the renal parenchyma (9). Nevertheless, some studies have shown no difference in oncologic outcomes between renal pelvic and ureteral tumors (10, 11).

Compared with renal pelvic tumors, ureteral tumors are associated with local recurrence of surgical bed recurrence (12). Adjuvant radiotherapy has been reported to decrease locoregional recurrence rates in patients with locally advanced UTUC (13). In addition, the POUT trial reported that adjuvant platinum-based chemotherapy administered within 90 days after RNU can significantly contribute to survival benefits in patients with UTUC (5). Thus, in our study, we aimed to investigate the prognostic impact of tumor location in patients with pT3N0M0 UTUC who may respond to adjuvant therapy after RNU.

## Materials and Methods

### Study Population

This study included patients with clinical T3N0M0 UTUC who underwent RNU at our institution between January 2005 and August 2019 and excluded patients who underwent nephron-sparing surgery and those with non-urothelial carcinoma histology. Finally, 302 patients with clinical and pathological data were included for the analysis. Among them, 136 (45.0%) and 166 (55.0%) patients underwent open and laparoscopic RNU. Clinical T3N0 was defined as image nodal negative UTUC. We selected the patients with clinically T3N0 UTUC. The lymph node (LN) dissection rate was 16.9% in this cohort because template LN dissection was not routinely performed in our institute. All patients underwent preoperative cystoscopy or computed tomography (CT) to determine the presence of a concurrent bladder tumor or distant metastasis. Demographic data such as age, sex, smoking history, concurrent bladder cancer, adjuvant therapy, disease recurrence outcome, and death were obtained using chart review. This study was approved by the institutional review board of our hospital (IRB number: 202000185B0), and written privacy consent was obtained from all participants.

### Pathological Evaluation

The diagnosis of UC was confirmed by histological analysis, and variant histology was also included in this study. Genitourinary pathologists reviewed all slides according to strict identical criteria and were blinded to the clinical outcomes. Tumor grading was evaluated according to the 2004 and 2016 World Health Organization classifications (14, 15). Tumors were staged according to the Eighth American Joint Committee on Cancer (AJCC) tumor–node–metastasis (TNM) classification. We excluded pT4 tumors of the renal pelvis that also infiltrated beyond the renal capsule into the perinephric fat. Renal pelvis tumors which invades through the basement membrane and into the renal parenchyma was staged as pT3. In addition, pagetoid spread of tumor within collecting ducts and renal tubules were excluded due to its CIS nature (16). Lymphovascular invasion, grade, variant histology, and concomitant carcinoma *in situ* were also assessed in each representative slide.

### Follow-Up Protocol and Definition of an Oncological Event

Our institutional follow-up protocol included postoperative fiber-cystoscopy every 3 months and renal ultrasonography to assess the contralateral urinary tract every 6 months during the first 2 years, every 6 months during the third year, and then annually thereafter. Abdominal CT was performed either annually or depending on the patient’s condition to assess lymph node status and local or regional recurrence of the tumor. Bone scanning, chest CT, and magnetic resonance imaging were performed when clinically indicated. Intravesical recurrence was defined as post-nephroureterectomy urinary bladder tumor recurrence. Local recurrence was defined as locoregional recurrence at the ipsilateral surgical field, and distant metastasis was defined as disease recurrence outside the urinary tract and out of the locoregional surgical field. Disease in the urinary bladder or contralateral upper urinary tract was not considered to indicate metastasis. Cancer-specific mortality was defined as local recurrence or distant metastasis at the time of death.

### Statistical Analysis

SPSS v.17 software was used for statistical analysis. Chi-square or two-sample t-tests were used to understand the distribution of these two groups and for intergroup comparisons. The Kaplan–Meier method with log-rank test was used to compare intravesical recurrence-free survival (IVRFS), local recurrence-free survival (LRFS), distant metastasis-free survival (DMFS), and cancer-specific survival (CSS) between the groups. Multivariate Cox regression analysis was used to identify independent prognostic factors upon oncologic outcome; only the parameters with p-value < 0.1 (borderline significance) in the univariate analysis were included in the multivariate analysis. Statistical significance was set at p < 0.05, as shown in **Table 1**, and an independent association was defined as p-value < 0.05, as shown in **Table 2**.

**Table 1:** Clinicopathologic Characteristics for pT3N0M0 UTUC.

Variables	Renal pelvic UC n = 191	Ureteral UC n = 111	*p* value
Follow up (Months)	45.1 ± 41.0	38.8 ± 34.0	0.156
Age (years)	68.2 ± 10.7	67.6 ± 10.0	0.625
Sex, n (%)			0.806
Male	97 (50.8%)	58 (52.3%)	
Female	94 (49.2%)	53 (47.7%)	
Nodal status, n (%)			0.475
Nx	156 (81.7%)	95 (85.6%)	
N0	35 (19.3%)	16 (14.4%)	
Smoking, n (%)	25 (13.1%)	15 (13.5%)	0.916
Concurrent Bladder cancer, n (%)	21 (11.0%)	15 (13.5%)	0.515
High grade, n (%)	190 (99.5%)	111 (100%)	0.445
Lymphovascular invasion, n (%)	105 (55.0%)	53 (47.7%)	0.225
Papillary, n (%)	137 (71.7%)	51 (45.9%)	<0.001
Carcinoma in situ, n (%)	86 (45.0%)	51 (45.9%)	0.877
Variant histology, n (%)	92 (48.2%)	30 (27.0%)	<0.001
Adjuvant chemotherapy, n (%)	21 (11.0%)	21 (18.9%)	0.055
5-year Intravesical recurrence, n (%)	37 (19.4%)	33 (29.7%)	0.055*
5-year Local recurrence, n (%)	43 (22.5%)	52 (46.8%)	<0.001*
5-year Distant metastasis, n (%)	54 (28.3%)	45 (40.5%)	0.073^*^
5-year Cancer death, n (%)	39 (20.4%)	31 (27.9%)	0.153^*^

*, Kaplan Meier analysis.

**Table 2:** Multivariate Cox proportional hazard regression analyses to predict oncological outcome from upper tract urothelial carcinoma after radical nephroureterectomy.

Variable	Intravesical Recurrence			Local Recurrence			Distant Metastasis			Cancer Specific Death		
	Uni	Multi	HR (95%CI)	Uni	Multi	HR (95%CI)	Uni	Multi	HR (95%CI)	Uni	Multi	HR (95%CI)
U *vs*. RP	0.055	0.074	1.54 (0.96-2.46)	<0.001	0.001	2.05 (1.33-3.15)	0.073	0.08	1.45 (0.96-2.20)	0.153		
Con. BCa	0.021	0.046	1.86 (1.01-3.41)	0.286			0.804			0.044	0.055	1.81 (0.99-3.30)
LVI	0.055	0.086	0.66 (0.41-1.06)	0.032	0.031	1.58 (1.04-2.38)	0.001	0.001	2.05 (1.35-3.09)	0.193		
LND	0.1			0.434			0.52			0.481		
PA	0.197			0.003	0.130	0.72 (0.47-1.10)	0.062	0.189	0.76 (0.50-1.15)	0.405		
CIS	0.770			0.343			0.671			0.239		
Variant	0.255			0.389			0.193			0.158		
HG	0.579			0.509			0.507			0.576		
Adj. CT	0.190			0.556			0.928			0.739		
Smoking	0.003	0.036	1.87 (1.04-3.37)	0.997			0.014	0.004	2.04 (1.25-3.33)	0.002	0.001	2.46 (1.43-4.24)
Female	0.293			0.908			0.498			0.311		
Age>68	0.731			0.993			0.014	0.003	1.84 (1.23-2.77)	0.047	0.020	1.77 (1.10-2.86)

Uni, Univariate; Multi, Multivariate; HR, Hazard ratio; CI, Confidence interval; U, ureter; RP, renal pelvis; BCa, Bladder cancer; LVI, Lymphovascular invasion; LND, Lymph node dissection; PA, Papillary; CIS, Carcinoma in situ; HG, High grade; Adj. CT, Adjuvant chemotherapy.

## Results

Patient clinicopathological characteristics stratified by tumor location are summarized in **Table 1**. Our cohort included 155 men (51.3%) and 147 women (48.7%). The median age of all patients was 68 years (interquartile range [IQR]: 62–76 years). The total median follow-up duration was 42.7 months (IQR: 13.4–62.9 months). Overall, 191 (63.2%) patients with renal pelvic tumors and 111 (36.8%) with ureteral urothelial carcinoma were analyzed. Renal pelvic tumors were more likely to be associated with papillary architecture than ureteral tumors (71.7% and 45.9%, respectively; p < 0.001). The presence of variant histology was more frequent in renal pelvic tumors (48.2%) than in ureteral tumors (27.0%) (p < 0.001). 35 (19.3%) patients with renal pelvic tumors and 16 (14.4%) patients with ureteral tumors received LN dissection during RNU (p= 0.475). No significant difference was found in the rest of the clinical features between the two groups (**Table 1**).

**Figure 1:**
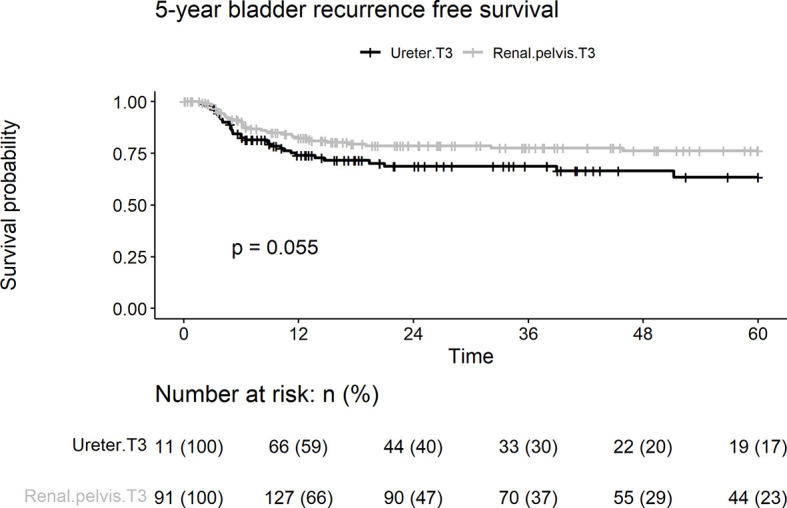
Kaplan-Meier analyses for 5-year intravesical recurrence-free survival stratified by tumor stage among patients with pT3N0M0 upper tract urothelial carcinoma following radical nephroureterectomy.

Overall, 70 patients (23.2%) experienced intravesical recurrence. Kaplan–Meier survival curves with a log-rank test stratified by tumor location showed that pT3 ureteral tumors were associated with borderline significantly lower IVRFS than were renal pelvic tumors (**Figure 1**; 5-year intravesical recurrence rates: 19.4 *vs*. 29.7%, p = 0.055). On univariate and multivariate analyses, concurrent bladder cancer and smoking were associated with intravesical recurrence (**Table 2**). Tumor location was a borderline risk factor for intravesical recurrence (adjusted hazard ratio [HR]: 1.54, ureter *vs*. renal pelvis; 95% confidence interval [CI], 0.96–2.46; p = 0.074).

**Figure 2:**
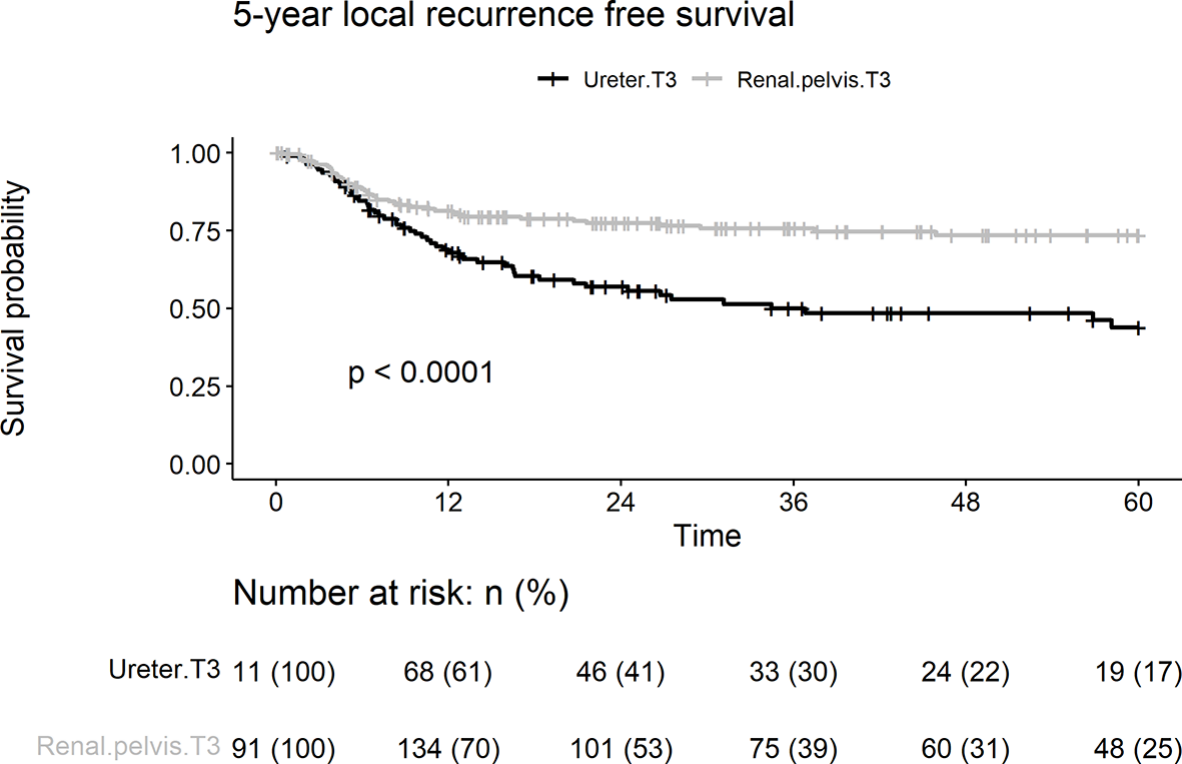
Kaplan-Meier analyses for 5-year local recurrence-free survival stratified by tumor stage among patients with pT3N0M0 upper tract urothelial carcinoma following radical nephroureterectomy.

In this cohort, 95 patients (31.5%) presented with local recurrence, including 43 (22.5%) and 52 (46.8%) patients with renal pelvic and ureteral UC, respectively. Patients with pT3 ureteral tumors had a significantly shorter LRFS duration than those with renal pelvic tumors (log-rank test, p < 0.001; **Figure 2**). Univariate analyses showed that tumor location, lymphovascular invasion, and papillary architecture (p < 0.001, p = 0.032, and p = 0.003, respectively) were significantly associated with local recurrence (**Table 2**). Multivariate analysis revealed that tumor location (HR = 2.05, p = 0.001) and lymphovascular invasion (HR = 1.58, p = 0.031) were independent risk factors for predicting local recurrence of UTUC.

**Figure 3:**
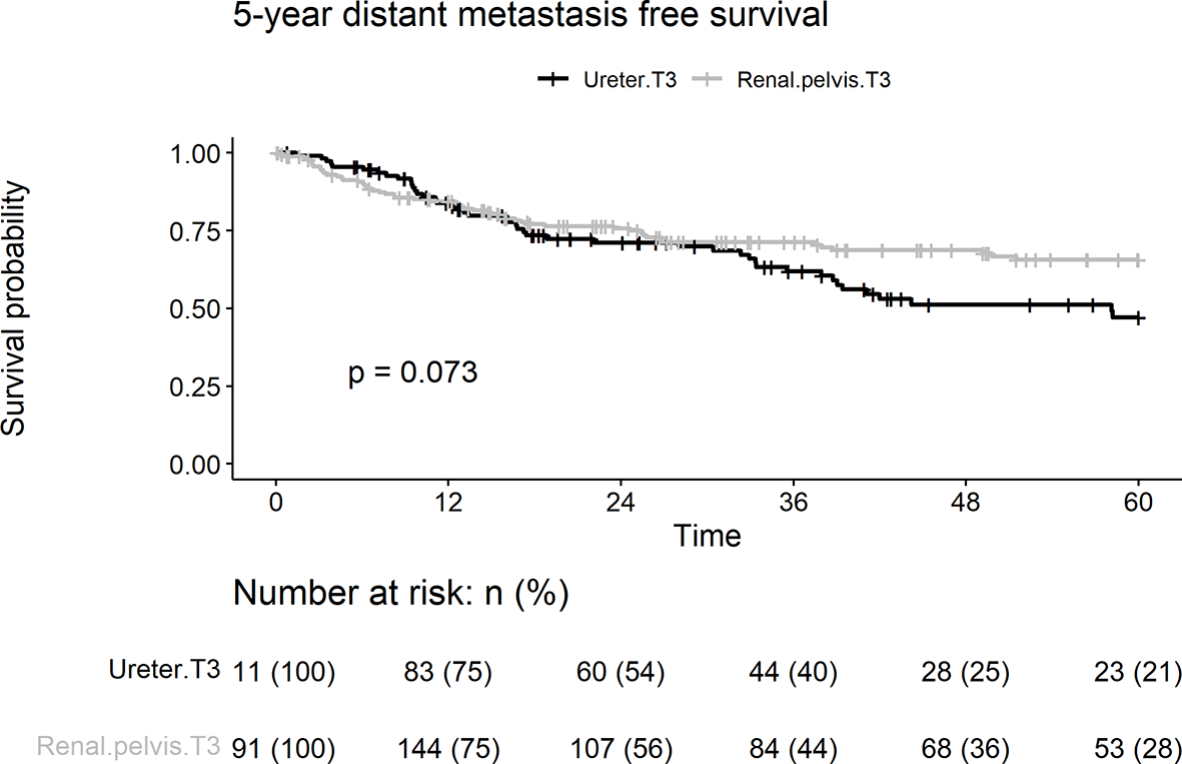
Kaplan-Meier analyses for 5-year distant metastasis-free survival stratified by tumor stage among patients with pT3N0M0 upper tract urothelial carcinoma following radical nephroureterectomy.

During follow-up, distant metastases were observed in 99 (32.8%) patients. The 5-year distant metastasis rate in the ureteral group (40.5%) was significantly higher than that in the renal pelvic group (28.3%; p = 0.073) (**Figure 3**). Lymphovascular invasion, smoking, and age > 68 years were associated with distant metastasis of UC on univariate and multivariate analyses (**Table 2**). Tumor location was a borderline independent risk factor for distant metastasis (adjusted hazard ratio [HR]: 1.45, ureter *vs*. renal pelvis; 95% confidence interval [CI], 0.96–2.20; p = 0.08).

**Figure 4:**
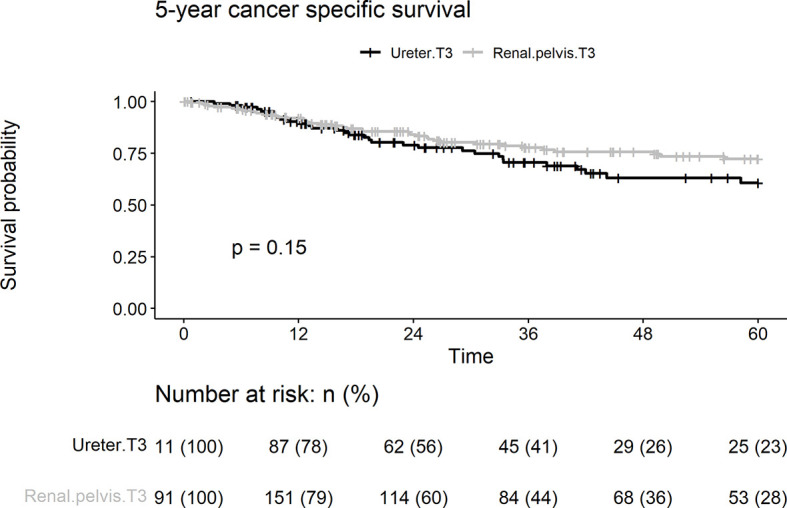
Kaplan-Meier analyses for 5-year cancer specific survival stratified by tumor stage among patients with pT3N0M0 upper tract urothelial carcinoma following radical nephroureterectomy.

Cancer-specific death from pT3 UTUC occurred in 70 (23.2%), 39 (20.4%), and 31 patients (27.9%) in the renal pelvic and ureteral groups, respectively. There were no differences between the two groups in terms of 5-year cancer death rates (p = 0.153) (**Figure 4**). On univariate analyses, age > 68 years, concurrent bladder cancer, and smoking were associated with cancer death (**Table 2**). On multivariable analysis, smoking and age > 68 years remained associated with worse cancer survival. Tumor location was not a risk factor for cancer death.

## Discussion

Radical nephroureterectomy with bladder cuff excision is the standard treatment for UTUC. Despite surgical management, the prognosis of UTUC remains poor. The 5-year cancer specific survival of advanced UTUC is 50% for T3 tumors and <20% for T4 tumors (17). Tumor stage, tumor architecture (18), tumor grade, and lymphovascular invasion have been identified as prognostic factors for UTUC. In addition to these well-established predictors, tumor location has been reported as a potential risk factor for oncological outcomes (2). Several studies have described that patients with ureteral tumors seem to be associated with a worse prognosis than patients with renal pelvis tumors. However, there remains a paucity of detailed research analyses of oncological outcomes in the literature. Although they were large population-based analyses, the authors of the aforementioned studies evaluated the impact of tumor location without focusing on locally advanced stage-specific and node-negative UTUC (7, 8, 19).

Although the recurrence rate of locally advanced UTUC is relatively high, variations in clinical course are often observed in clinical practice (20). Adjuvant therapy is a common treatment strategy used to improve survival rates in this population (5, 21). According to the reviewed literature, there is a scarcity of data focused on the prognostic impact of tumor location in patients with locally advanced UTUC. In the present study, we demonstrated that pT3 ureteral tumor location was independently associated with local recurrence and borderline associated with intravesical recurrence and distant metastasis.

The disease recurrence of UTUC include bladder, local, and distant recurrence, with the majority of relapses occurring within the first 3 years after RNU (22). Early studies have reported that anatomical reasons may result in prognostic differences. Park et al. found that ureter UC was associated with significantly higher disease recurrence and cancer death. They supposed that the renal pelvis is located within the perinephric fat, which may serve as a barrier to resist metastatic spreading. However, the ureter is surrounded by weak adipose tissues surrounded by thinner adventitia that are rich in blood plexus and lymphatic ducts, facilitating tumor invasion (23). An additional anatomical difference is regarding the thinness of the muscularis of propria. Muscularis propria may be entirely absent in the renal pelvis with only a thin layer of fibrous connective tissue between the urothelium and the kidney. Therefore, pT3 tumors in renal pelvis may occur earlier compared to the ureter tumors (16). Thus, it is a challenge for surgeons to completely excise ureteral tumors and adjacent tissues, which may contain micro-metastases. Among renal pelvic tumors with peripelvic adipose tissues invasion, the distinction between T3 and T4 depends on the presence of Gerota’s fascia. Ureter tumor located in periureteral fat is regarded as T3 disease unless it invades adjacent organs which occurs very rarely (pT4). Given that the limitation of distinction between T3 and T4 by surgical resection and ureter anatomy, surgical margins may help to identify tumors with high risk of local recurrence. Yoo et al. found that tumor location was associated with local recurrence of UTUC after RNU, and ureteral UC was a significant risk factor for surgical bed recurrence among 353 patients with UTUC. However, this study was not stage-specific. In addition, most patients had T2 or less (75.9%) and low-grade UTUC (55%) (12). In addition, local recurrence may be related to tumor spillage during surgery and lymph node status. In our study, we selected patients with clinically T3N0 UTUC and performed RNU according to oncological principles preventing tumor seeding. Over time, minimally invasive approaches are increasingly being utilized with less perioperative complications and faster recovery (24). Lenis et al. concluded that compared with open RNU, robot-assisted RNU does not compromise performance of lymph node dissection and may be associated with LN yield (25).

Adjuvant treatment such as radiotherapy may be considered for patients at high risk of local recurrence. A Korean multicenter study demonstrated that adjuvant radiation therapy significantly reduced the local recurrence in patients with advanced-stage, nonmetastatic UTUC (21). In this cohort, we found that tumor location had a more significant impact on local recurrence than lymphovascular invasion. The high local recur rate of ureter cancer was the clinical unmet need that we observed. Adjuvant radiation therapy may be considered according to recurrence pattern to improve the local regional control rate. Furthermore, well-known risk factors for local recurrence can help clinicians in risk stratification, patient selection for adjuvant radiotherapy, and active surveillance.

The incidence of intravesical recurrence in patients with UTUC after RNU ranges from 22% to 47% (26–28). Tumor location was also a controversial risk factor for intravesical recurrence in previous studies. In this cohort, ureteral UC presented with borderline independent significance for intravesical recurrence. In a propensity score-matched case-control study published in 2020, Jiang et al. evaluated 229 patients with UTUC and demonstrated that tumor location was not an independent predictor for intravesical recurrence, where tumors were classified as located in either the renal pelvis or the ureter (29). However, Otsuka et al. reported that a lower ureteral lesion served as an independent risk factor for intravesical recurrence, where a lower ureter lesion was defined as the lowest cancer component within 5 cm from the lower end of the ureter (30). This difference in oncologic outcomes may be related to the different classification methods of tumor position. Delayed detection of intravesical recurrence might cause the development of muscle invasive disease and impaired quality of life in patients with UTUC. Therefore, intensive surveillance of intravesical recurrence is necessary.

Smoking is a modifiable risk factor of bladder cancer (31). Furthermore, Rink et al. reported that smoking may increase the risk of disease recurrence and cancer-specific mortality in patients with UTUC (32). We found that old age, smoking, and lymphovascular invasion were independent prognostic factors for UTUC in distant metastasis. However, tumor location was found to be a borderline independent risk factor for distant metastasis. For locally advanced UTUC, the most recent and largest randomized controlled study (the POUT trial) indicated that adjuvant chemotherapy improved survival after RNU in patients in whom systemic chemotherapy was not contraindicated (5). Nevertheless, adjuvant chemotherapy was not associated with prognosis in this cohort. This result may be attributed to the fact that not all patients were eligible for chemotherapy or an incomplete treatment cycle.

To our knowledge, there are a number of limitations to the present study. First, this was a retrospective study conducted in a single institution, which may have some intrinsic bias. Second, the prognostic impact was found for the first intravesical recurrence, local recurrence, and distant metastasis, rather than cancer-specific mortality. However, this method was the most objective way to observe cancer behavior due to the presence of confounding factors such as a sequential treatment strategy, which depends on each patient’s socioeconomic status, hospice care choice, and general health condition. Third, patients who received adjuvant chemotherapy did not always fulfill the standard treatment cycles due to adverse effects and patient preferences (5). In addition, follow-up images such as abdominal/chest CT, MRI or bone scanning were arranged based on patient’s condition rather than a standard protocol. Disease recurrence may be undetected. The low rate of LN dissection (16.9%) also could be a bias and influenced our results independently of surgical approach, since local recurrence could be related to LN recurrence in the ipsilateral surgical field. We showed that ureteral T3 tumors were independently associated with local regional recurrence after RNU. This result could help guide decision-making about more intensive postoperative adjuvant chemotherapy or even radiation to improve local regional disease control and might prevent further distant metastasis.

In conclusion, ureteral tumor location was an independent risk factor for local recurrence and borderline independent risk for intravesical recurrence and distant metastasis in patients with pT3N0M0 UTUC following RNU. Local recurrence differences could be influenced by different level of surrounding tissues invasion classified as pT3 such as Gerota’s fascia and renal parenchyma that are absent in ureteral tumors. In addition, regular postoperative follow-up and feasible adjuvant therapies should be considered to improve the oncological outcomes. Further studies are necessary to validate the tumor location-specific clinical cancer course. According to our observation, a prospective adjuvant trial focused on local regional control and prevention of subsequent distant metastasis for ureter T3 tumors is worthy of investigation to improve the oncological outcome.

## Data Availability Statement

The original contributions presented in the study are included in the article/supplementary material. Further inquiries can be directed to the corresponding author.

## Author Contributions

TC: manuscript draft and analysis. HLL: study design, analysis and supervision. HW, PC, WY, YCC, WChinL, YTChen, YTCheng, CK, WChiaL, CC, YCS, YL, HYL, YC, YLS, and CH: data collection, administrative, technical, and material support. All authors contributed to the article and approved the submitted version.

## Funding

This study was supported by grants from Chang Gung Memorial Hospital Research Project Grant CMRPG8K1091, CMRPG8K1092.

## Conflict of Interest

The authors declare that the research was conducted in the absence of any commercial or financial relationships that could be construed as a potential conflict of interest.

## Publisher’s Note

All claims expressed in this article are solely those of the authors and do not necessarily represent those of their affiliated organizations, or those of the publisher, the editors and the reviewers. Any product that may be evaluated in this article, or claim that may be made by its manufacturer, is not guaranteed or endorsed by the publisher.
